# Epistaxis of Alarming Character Arrested by Injections of Perchloride of Iron

**Published:** 1860-02

**Authors:** E. J. Fountain

**Affiliations:** Davenport, Iowa


Severe Hæmorrhage from the Tonsil arrested by the solution of the Perchloride of Iron.—The following case of severe hæmorrhage admirably illustrates the beneficial effect of the perchloride of iron as a stypic. Mr. Henry Thompson was called to see it at the Marylebone Infirmary, at midnight, on the 22d ult., and took with him a bottle of the solution of the perchloride of iron (*French Codex*) and a glass brush. He found a woman propped up in bed, pale and almost pulseless, with a large vessel of coagulated blood at her side, and the bedclothes bespattered in every direction. She could not speak so as to be understood, and finding that she was bleeding from one or both tonsils, Mr. Thompson passed his finger into the fauces and discovered a small opening, as if from an incision previously made, in the front of the right tonsil; blood was evidently flowing fast from it. Binding a piece of lint round the entire left forefinger, so as to form a mop, pressure was kept upon the wound, until he could apply the solution, which was done freely two or three times. This at once arrested the bleeding, which recommenced in a quarter of an hour, and was again stopped by the same plan. There was no return after this, and she is now recovering rapidly.

In default of the aid thus rendered by the stypic solution, Mr. Thompson had made up his mind to tie the caroted artery; but the efficacy of the treatment was so striking, that the operation was not required.—*London Lancet.*

---

Epistaxis of alarming character arrested by Injections of the Perchloride of Iron.—*By E. J. Fountain, M. D., Davenport, Iowa.*—I am induced to publish the following case from reading the report of "Death from Epistaxis," occurring in the practice of Dr. Triplett, of Virginia.

About two years ago, I was summoned in haste to see a young man who was reported to be in a dying condition from loss of blood. The bleeding had continued uninterruptedly for about thirty hours, escaping constantly from the nostrils, and frequently thrown out in clots from the posterior nares.

He had been attended by a German physician, who had not succeeded in arresting the hæmorrhage, and before my arrival he had abandoned the case, from motives which I need not here mention.

I found him in a frightful condition, his face, hands, linen, and much of the bedclothing, and the floor, being covered with blood. In one corner of the miserable apartment, where many people were crowded, was a pile of rags and towels, saturated with blood.

He was so impoverished that he could not support himself in an upright position, and the extreme pallor of his skin and colorless lips indicated plainly that he had lost a large quantity of blood.

I immediately plugged the nostrils, anteriorly and posteriorly, in the usual way. I supposed this would arrest the hæmorrhage, but I was mistaken. Very soon the blood escaped through the plugs in each direction, and the flow returned as profuse as before. I then removed the plugs, and rolling up two quite large pieces of fine, dry sponge, I introduced them as before, and so firmly, that I thought it would be impossible for a drop of blood to escape. I then left him, and returning an hour after, I was astonished to find the blood escaping as freely as ever. I again removed the plugs, and washing out the nostrils by injections of cold water, I pressed into each a roll of *tannin*, made into a soft mass with a little water and glycerine; I packed the nostrils full of this, but it did no good. I then introduced the plugs a third time, using compressed sponge, and forcing them in so firmly, that I feared I might have great difficulty in removing them. I then had ice applied constantly to either side of the nares, and kept the patient perfectly quiet. This answered the purpose for about half an hour, and then the bleeding returned as bad as ever. By this time the patient experienced the alarming symptoms of excessive loss of blood—ringing in the ears, occasional blindness, etc. The case looked desperate to me, while the patient, and even some of his friends, protested against further effort, as useless and cruel. I saw that it *must* be checked very soon, or it would surely be fatal. I removed the plugs with some difficulty, and washing out the nares, I passed into each a piece of nitrate of silver, about a quarter of an inch in length, carrying them back with the forceps about four inches. I also introduced a curved injecting instrument, perforated towards its extremity with a number of small openings, and forcibly injected a strong solution of the same caustic. This did no good.

It now occurred to me that the perchloride of iron might answer the purpose. I soon procured it, and after washing out the nostrils as before, I injected a quantity of the undiluted perchloride into each nostril. This immediately checked the bleeding, and proved an effectual remedy.

Twenty-four hours after this the bleeding again returned, when I repeated the injections, and with the same happy effect.

There was no recurrence of hæmorrhage after this, and the patient soon recovered so as to go about, but for quite a long time, felt the effects of the loss of so much blood, which had been so excessive as to leave him but a very slender hold upon life. I know not in what other way it could have been saved; and the publication of this may be the means of rescuing others in similar circumstances.—*Amer. Med. Monthly.*

